# Ki-67 as a prognostic marker in early-stage non-small cell lung cancer in Asian patients: a meta-analysis of published studies involving 32 studies

**DOI:** 10.1186/s12885-015-1524-2

**Published:** 2015-07-15

**Authors:** Song Wen, Wei Zhou, Chun-ming Li, Juan Hu, Xiao-ming Hu, Ping Chen, Guo-liang Shao, Wu-hua Guo

**Affiliations:** 1 Department of Interventional Radiology, Zhejiang tumor hospital, Hangzhou, 310022 China; 2 Interventional Room of Oncology, Second Affiliated Hospital, Nanchang University, Nanchang, 330006 China; 3 Department of Vascular Surgery, Second Affiliated Hospital, Nanchang University, Nanchang, 330006 China; 4 Department of Dermatology, Second Affiliated Hospital, Nanchang University, Nanchang, 330006 China; 5 Department of Medical, Second Affiliated Hospital, Nanchang University, Nanchang, 330006 China; 6 Department of Gastroenterology, Second Affiliated Hospital, Nanchang University, Nanchang, 330006 China

**Keywords:** Ki-67, Meta-analysis, Non-small cell lung cancer, Prognostic value

## Abstract

**Background:**

Despite the large number of published papers analyzing the prognostic role of Ki-67 in NSCLC, it is still not considered an established factor for routine use in clinical practice. The present meta-analysis summarizes and analyses the associations between Ki-67 expression and clinical outcome in NSCLC patients.

**Methods:**

PubMed, Cochrane, and Embase databases were searched systematically using identical search strategies. The impacts of Ki-67 expression on survival in patients with NSCLC and NSCLC subtypes were evaluated. Furthermore, the association between Ki-67 expression and the clinicopathological features of NSCLC were evaluated.

**Results:**

In total, 32 studies from 30 articles met the inclusion criteria, involving 5600 patients. Meta-analysis results suggested that high Ki-67 expression was negatively associated with overall survival (OS; HR = 1.59, 95 % CI 1.35-1.88, *P* < 0.001) and disease-free survival (DFS; HR = 2.21, 95 % CI 1.43-3.42, *P* < 0.001) in NSCLC patients. Analysis of the different subgroups of NSCLC suggested that the negative association between high Ki-67 expression and OS and DFS in Asian NSCLC patients was stronger than that in non-Asian NSCLC patients, particularly in early-stage (Stage I-II) adenocarcinoma (ADC) patients. Additionally, while high expression was more common in males, smokers, and those with poorer differentiation, there was no correlation between high Ki-67 expression and age or lymph node status. Importantly, significant correlations between high Ki-67 expression and clinicopathological features (males, higher tumor stage, poor differentiation) were seen only in Asian NSCLC patients.

**Conclusions:**

The present meta-analysis indicated that elevated Ki-67 expression was associated with a poorer outcome in NSCLC patients, particularly in early-stage Asian ADC patients. Studies with larger numbers of patients are needed to validate our findings.

**Electronic supplementary material:**

The online version of this article (doi:10.1186/s12885-015-1524-2) contains supplementary material, which is available to authorized users.

## Background

Lung cancer (LC) is often fatal and is very common worldwide. It has been reported that the overall 5-year survival rate of lung cancer patients was ~16 %, and that it was < 70 % even in patients diagnosed at stage I [1]. Non-small cell lung cancer (NSCLC), of which adenocarcinoma (ADC) and non-ADC (including squamous cell carcinoma (SQCC), large cell lung carcinoma (LCC), and bronchial gland carcinoma (BGC)) account for the majority of cases, represents almost 80 % of primary LC cases. Although the treatment of LC is becoming more individualized, there is an urgent need for reliable prognostic factors to predict clinical outcome and to more precisely stratify the group of patients with poorer outcomes.

Ki-67 is expressed in proliferating cells and has been used in clinical practice as an index to evaluate proliferative activity in NSCLC and other cancers [2, 3]. Moreover, several studies have suggested that high Ki-67 expression in a tumor is a strong prognostic factor in NSCLC [4–7]. However, despite the large number of published papers analyzing the prognostic role of Ki-67 in NSCLC, it is still not considered an established factor for routine use in clinical practice [8, 9]. Although a large meta-analysis involving 17 studies published in 2004 showed that high expression of Ki-67 *was* associated with a poorer overall survival (hazard ratio (HR) 1.56, 95 % confidence interval (CI) 1.30–1.87), it did not evaluate the association between Ki-67 expression and disease-free survival. Most importantly, because of the limited number of studies and patients included, it did not examine high Ki-67 expression in *Asian* patients [2]. Thus, a further meta-analysis investigation is needed to delineate the relationship between Ki-67 expression and prognostic significance in NSCLC more clearly.

In this study, we performed a meta-analysis to explore the relationship between Ki-67 expression and its prognostic value in NSCLC. Associations between Ki-67 expression and the clinicopathological features of NSCLC, including age, gender, smoking status, lymph node status, and tumor differentiation, were also evaluated.

## Methods

The protocol, including the objective of our analysis, criteria for study inclusion/exclusion, assessment of study quality, primary outcome, and statistical methods, was in accordance with the Preferred Reporting Items for Systematic Reviews and Meta-Analyses (“PRISMA”) statement (Additional files 1 and 2) [10].

### Study selection

The PubMed, Cochrane, and Embase databases were searched systematically for relevant articles published up to November 1, 2014. Search terms included Non-Small-Cell Lung Cancer (‘Carcinoma, Non-Small-Cell Lung’ or ‘Carcinoma, Non Small Cell Lung’ or ‘Carcinomas, Non-Small-Cell Lung’ or ‘Lung Carcinoma, Non-Small-Cell’ or ‘Lung Carcinomas, Non-Small-Cell’ or ‘Non-Small-Cell Lung Carcinomas’ or ‘Carcinoma, Non-Small Cell Lung’ or ‘Non-Small-Cell Lung Carcinoma’ or ‘Non Small Cell Lung Carcinoma’ or ‘NSCLC’), Ki-67 (‘Ki-67’ or ‘Ki67’ or ‘MIB-1’ or ‘MIB 1’ or ‘proliferative index’), prognosis, survival, and outcome, in all possible combinations. Using these parameters, we filtered out all the eligible articles and looked through their reference lists for additional studies. The systematic literature search was undertaken independently by two reviewers (SW and ZW) and ended in November 2014. Disagreements were determined through consensus with a third reviewer (CL). Authors of the eligible studies were contacted for additional data relevant to this meta-analysis, as necessary.

### Inclusion and exclusion criteria

Inclusion criteria for the primary studies were 1) inclusion of patients with a distinct NSCLC diagnosis by pathology, 2) measurement of Ki-67 expression using immunohistochemistry (IHC) in primary NSCLC tissue, 3) investigation of the relationship between Ki-67 expression and overall survival (OS) or disease-free survival (DFS) in patients with NSCLC and availability of valid survival data either provided directly or that could be calculated indirectly, and 4) publication in the English language. When authors had several publications or reported on the same patient population, only the most recent or complete study was included.

Exclusion criteria for the primary studies were 1) an overlap among articles or duplicate data; 2) the use of animals or cell lines; 3) insufficient data availability for estimating HR and 95 % CI, such as typical of abstracts, editorials, letters, conferences data, expert opinions, reviews, and case reports; 4) investigation of the relationship between Ki-67 and NSCLC using methods other than IHC; 5) inclusion of patients who underwent chemotherapy or radiotherapy interventions; and 6) a study sample comprising fewer than 20 patients.

### Data extraction and literature quality assessment

Two investigators (SW and WZ) conducted the data extractions independently [10]. Any discrepancies were determined by reviewing the articles together until a consensus was reached. The following information was extracted from each article: name of first author and publication date; study population characteristics such as number of patients, age, gender, and treatment during follow-up; tumor data such as pathology, type of NSCLC, Ki-67 expression in the primary site, and TNM stage; variables such as tissue Ki-67 measurement method, cut-off value for the Ki-67 level; survival data, such as OS and DFS; and relevant quality scores. The primary data were the HR and 95 % CI for survival outcomes, including OS and DFS.

For study quality control, we used the Reporting Recommendations for Tumor Marker Prognostic Studies (REMARK) and extracted 18 items (Additional file 3: Table S1). Each item was scored on a scale of 0–2, with 2 indicating a complete description, 1 indicating a partly matched description, and 0 indicating no matched description. The maximum score was 36 [11, 12]. Any discrepancies were resolved by a consensus discussion with a third reviewer (CL).

### Statistical analysis

ORs with 95 % CIs were used to estimate the association between Ki-67 expression and the clinical characteristics of NSCLC patients, including age, gender, smoking habits, pathological type, TNM stage, tumor stage, lymph nodes status, and tumor differentiation status. According to the clinical characteristics, stages III and IV together were defined as ‘advanced stage’ and stages I and II as ‘early stage’. T_2_, T_3_, and T_4_ were all defined as ‘advanced stage’ compared with T_1_. N_1_, N_2_, and N_3_, which were combined into one group. Moderately and poorly differentiated were also combined [13, 14].

To identify the prognostic effect of Ki-67 expression, the overall HR and 95 % CI were evaluated for elevated Ki-67 expression. The combined ORs and HRs were initially estimated graphically using forest plots. Subgroup analyses were then conducted when the risk (OR or HR) was significant (*P* < 0.05).

The heterogeneity of the studies was assessed using Cochran’s Q test and the Higgins I^2^ statistic. When the I^2^ was below 50 %, the studies were considered to have acceptable heterogeneity, and a fixed effects model was used; otherwise, a random effect model was used.

To assess the stability of the results, we performed a sensitivity analysis in which one study at a time was removed to examine its individual influence on the pooled HR. Publication bias was evaluated using a funnel plot with Egger’s and Begg’s tests. *P* values < 0.05 were considered to indicate statistically significant publication bias. Additionally, ‘trim and fill’ analyses were used to evaluate the stability of our meta-analysis results if the plots were asymmetric [15]. All analyses were performed using the STATA software (er. 12.0; Stata Corp., College Station, TX, USA).

## Results

### Literature search and study characteristics

**Fig. 1:**
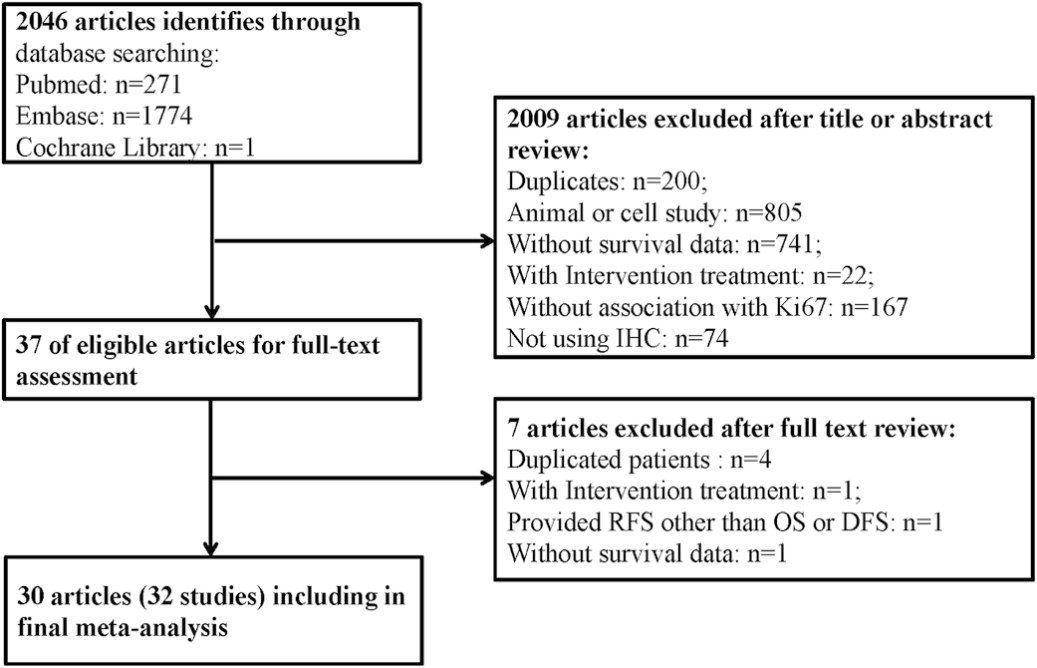
Flow diagram of the relevant studies selection procedure

We identified 2046 potentially relevant articles through the search strategy described in Methods. As shown in Fig. 1, 2009 articles were excluded after the first screening based on the abstracts and/or titles, and 37 articles remained after reviewing their full texts for relevance. Seven articles were ultimately excluded, due to overlap with previously reported studies (*n* = 4) [16–19], use of interventional treatments (*n* = 1) [20], a lack of survival data (*n* = 1) [21], or providing RFS other than OS/DFS in NSCLC (*n* = 1) [22]. Additionally, two of the articles could be divided into two studies [23, 24]. Thus, a total of 30 eligible articles [5–9, 23–47] involving 32 studies were included in this meta-analysis. The flow diagram of the study selection procedure is presented in Fig. 1.

As demonstrated in Table 1, 5600 patients with related clinical data from a total of 6178 patients were enrolled in the 32 studies, which were published between 1993 and 2014. All 32 studies were retrospective. Of the 32 studies, 11 were conducted in Japan, five in America, four in China, four in Italy, two in Canada, two in Korea, and one each in Argentina, Brazil, the Czech Republic, and Germany. The case size of each study varied from 44 to 494 (median, 156) patients. The age of the patients ranged from 19 to 89, and the overall proportion of males was 66.11 %.

**Table 1:** Characteristics of studies included in the final meta-analysis of Ki-67 expression and prognosis of NSCLC

First-Author and Year	Country	Total Patients, H/L	Mean age	Gender (M/F)	History	TNM Stage	Antibody and dilution	Cut-off (%)	Followup (median Month)	Survival Analysis, year	HR estimated	OS/DFS HR (95%CI)	Study Quality
Ahn 2014	Korea	109,20/89	65	65/44	NSCLC	I-III	Anti-Ki67; 1:50	40	30	OS/DFS,5	S.urves	O:1.60(0.74-3.44) D:2.875(1.326-6.234)	34
Cagini 2000x	Italy	99,43/56	66	91/8	NSCLC	I-II	MIB-1; 1:100	20	41	OS, 5	Events	O:1.33(0.72-2.43)	31
D’amico 1999	USA	408,204/204	62.9	269/139	NSCLC	I	MIB-1, NA	7	60	OS,5	Events	O:1.41(0.99-2.00)	33
Demarchi 2000	Brazil	64,32/32	59.8	43/21	ADC	I-III	MIB-1; 1:400	22.2	51.9	OS,5	R	O:0.49(0.20-1.22)	31
Fontanini 1996	Italy	65,31/34	46	63/7	NSCLC	I-III	MIB-1; 1:200	30.2	45	OS,3	R	O:1.05(0.83-1.324)	34
Haga 2003	Japan	187,112/75	NA	120/67	ADC,SCC	I	MIB-1; 1:100	10	120	OS,5	Events	O:3.636(1.267-10.439)	33
Harpole 1996	USA	275,109/106	63	177/98	NSCLC	I	Anti-Ki67; NA	7	68	OS, 5	Events	O:1.53(1.00-2.37)	34
Hayashi 2001	Japan	98,36/62	62.7	56/42	ADC	I-III	MIB-1; 1:200	12.6	60	OS,5	R	O:2.0(1.1-3.8)	29
Hommura 2000	Japan	215,116/99	63.3	144/71	NSCLC	I-IV	MIB-1; 1:50	30	84	OS,3	R	O:2.53(1.35-4.72)	34
Huang 2005	Japan	173,117/56	67	116/57	NSCLC	I-III	MIB-1; 1:40	25	77	OS,5	Events	O:1.56(0.99-2.44)	32
Ishida 1997	Japan	114,57/57	64.9	59/55	ADC	I-II	MIB-1; 1:50	22.7	28.5	OS,5	S.urves	O:8.50(3.52-20.53)	32
Kaira 2008	Japan	361,186/135	67	196/125	NSCLC	I-III	MIB-1; 1:40	25	48	OS,5	R	O:0.667(0.271-1.643)	32
Liu 2012a	China	494,113/381	61	366/128	ADC,SCC	I-IV	Anti-Ki67; 1:200	50	25.9	OS,5	R	O:1.583(1.100-2.277)	32
Liu 2012b	China	174,88/79	60	133/41	ADC,SCC	I-IV	Anti-Ki67; 1:200	50	25	OS,5	R	O:1.681(0.487-5.797)	32
Maddau 2006	Italy	180,103/77	65.5,	151/29	NSCLC	I-III	MIB-1; 1:50	25	3-47	OS,3	R	O:0.79(0.55-1.15)	29
Mehdi 1998	USA	243,154/49	63.5	184/76	NSCLC	I-II	MIB-1; 1:150	25	60	OS/DFS,3	S.urves	O:1.60(1.06-2.41) D:1.58(1.06-2.41)	36
Minami 2002	Japan	47,22/25	64	28/19	ADC	I	MIB-1; NA	20	89	OS,5	R	O:1.022(0.96-1.08)	33
Navaratnam 2012a	Canada	79,37/42	69.2	47/32	NSCLC	I-II	MIB-1; 1:50	20	36	OS,3	R	O: 1.81(0.93-3.53)	30
Navaratnam 2012b	Cadana	58,20/38	62.8	23/35	NSCLC	I-II	MIB-1; 1:50	10	36	OS,3	R	O:1.31(0.68-2.52)	24
Nguyen 2000	Czech	89,34/55	60	73/16	NSCLC	I-IV	MIB-1; NA	30	36	OS,3	S.urves	O:2.15(1.21-3.78)	28
Pence 1993	USA	61,15/46	63	56/5	NSCLC	I-IV	Anti-Ki67; 1:100	PI 3.5	38	OS,5	S. urves	O:2.18(1.00-4.78)	29
Poleri 2003	Argentina	50,28/22	60.8	NA	ADC,SCC	I	MIB-1; NA	33	59	DFS,5	Events	D:4.10(1.98-8.46)	33
Puglisi 2002	Italy	81,28/53	62.5	NA	ADC,SCC	I-III	MIB-1; 1:100	34.2	115.76	OS,5	R	O: 1.29(0.71-2.31)	33
Ramnath 2001	USA	212,118/94	63.7	111/101	NSCLC	I-IV	MIB-1; 1:100	25	24.3	OS,3	S. Curve	O:1.41(0.93-2.12)	31
Shiba 2000	Japan	156,81/75	62.4	112/44	NSCLC	I-III	MIB-1; 1:100	20	49	OS,5	S. Curve	O:2.20(1.38-3.53)	34
Takahashi 2002	Japan	62,22/40	66.9	40/22	ADC,SCC	I-II	MIB-1; 1:100	25	3.9	DFS,5	R	D:1.02(0.32-3.30)	33
Warth 2014	Germany	482,230/252	63.2	NA	ADC	I-IV	MIB-1, 1:500	25	45.6	OS/DFS,5	S. Curve	O:1.86(1.29-2.69) D:1.29(1.02-1.64)	29
Woo 2009	Japan	184,79/105	67.8	92/92	NSCLC	I	MIB-1; NA	10	35.9	DFS,5	R	D:3.84(1.18-12.45)	34
Wu 2013	China	192,120/72	59	104/88	NSCLC	I-III	Anti-Ki67; 1:200	10	60	OS/DFS,5	R	O:2.829(1.26-4.525) D:2.929(2.184-4.928)	32
Yamashita 2011	Japan	44,13/31	NA	25/19	NSCLC	I	Anti-Ki67; 1:100	5	60	DFS,5	R	D:12.5(1.1-140.7)	33
Yoo 2007	Korea	219,17/209	65.8	168/51	ADC,SCC	I-III	Anti-Ki67; NA	30	38.9	OS,5	R	O:0.827(0.319-2.140)	36
Zhong 2014	China	270,66/204	62	192/78	ADC,SCC	I-III	Anti-Ki67; 1:200	50	60	OS,5	R	O:2.179(1.096-4.333)	34

*Abbreviation:* HR hazard ratio, CI confidence interval, OS overall survival, DFS disease-free survival, NSCLC non-small-cell Lung cancer, ADC adenocarcinoma, SCC squamous carcinoma, R Author reported, O, OS, D,DFS, H High expression, L Low expression, S. curve Survival curve

All studies included information on disease stage, and the proportion of stages I + II was 67.9 %. IHC was the only technique used to detect Ki-67 expression, using various antibodies and cut-off values (range, 5–50 %), and 2503 (44.70 %) tissue samples had ‘high’ Ki-67 expression (Table 1).

Of the 32 studies, 19 provided HR and 95 % CI values directly, whereas in the other 13 studies, they were calculated from available data (*n* = 6) or from Kaplan–Meier survival curves (*n* = 7), as described by Tierney [48]. Of the 32 studies, 20 identified high Ki-67 expression as an indicator of poor prognosis, whereas the remaining 12 studies showed no significant effect of high Ki-67 expression on survival outcome.

### Methodological quality of the studies

The results of the quality assessment of the included studies are shown in Table 1. Quality scores ranged from 24 to 36, with a median value of 33. All of the studies satisfied most of the items and reported totals for the assay methods and confounders.

### Correlation of high Ki-67 expression with OS in NSCLC

**Fig. 2:**
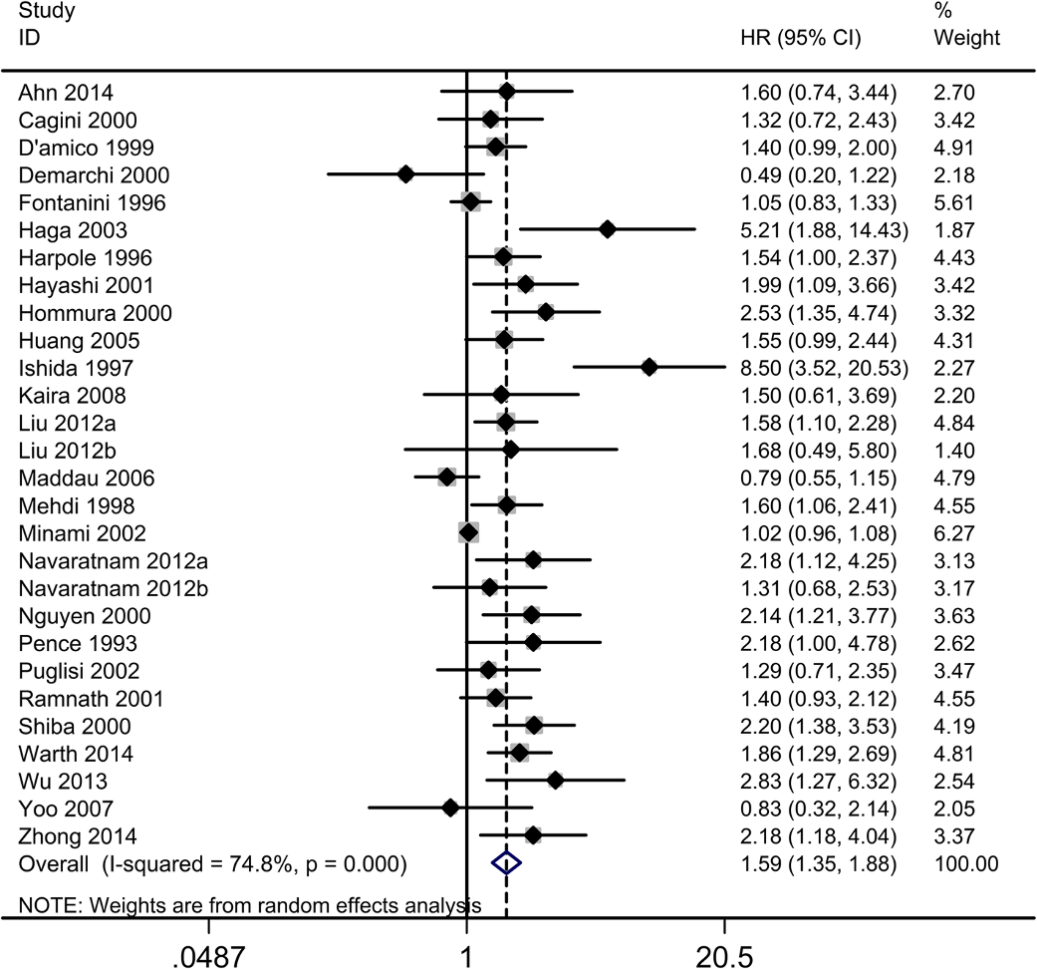
The hazard ratio (HR) of Ki-67 expression associated with OS in all NSCLC patients. HR > 1 implied worse OS for the group with high Ki-67 expression

Of the 28 studies investigating the association between Ki-67 expression and OS, 14 involved Asian patients (*n* = 2729) and 14 involved non-Asian patients (*n* = 2287). The overall HR and 95 % CI for NSCLC patients was 1.59 (95 % CI 1.35–1.88, *P* < 0.001, *n* = 5007), with significant heterogeneity (I^2^ = 74.8 %, *P* < 0.001; Fig. 2, Table 2). Subgroup analyses showed that the risk was significant in both Asian and non-Asian patients (HR 1.97, 95 % CI 1.43–2.71, *P* < 0.001 and HR 1.37, 95 % CI 1.15–1.64, *P* = 0.013, respectively) with significant heterogeneity (I^2^ = 82.1 %, *P* < 0.001 and I^2^ = 74.0 %, *P* < 0.001, respectively).

**Table 2:** HR values of OS and DFS of NSCLC subgroups

	Outcome	Studies (n)	Patients	HR	95%CI	P value	Model	H, I^2^, P value
**OS**	**All study**	28	4534	1.58	1.33-1.87	*0.000*	Random	100.02,74.0 %,0.000
	**Asian**	14	2729	1.97	1.43-2.71	*0.000*	Random	72.62,82.1 %,0.000
	**Non-Asian**	14	2278	1.37	1.15-1.64	*0.013*	Random	22.99,74.0 %,0.000
	**Stage I**	8	1144	1.85	1.27-2.69	*0.001*	Random	32.90,78.7 %,0.000
	**Stage I-II**	8	1166	1.72	1.20-2.46	*0.003*	Random	29.43,76.2 %,0.000
	**Stage I-III**	7	1038	1.60	1.21-2.12	*0.001*	Fixed	9.44, 36.5 %,0.150
	**Stage III-IV**	1	58	1.31	0.68-2.53	0.42	Fixed	-
	**ADC**	10	1327	2.21	1.38-3.50	*0.000*	Random	64.38,86.0 %,0.000
	**Asian**	6	666	3.01	1.96-4.02	*0.000*	Random	8.70,42.5 %,0.122
	**Non-Asian**	4	661	1.31	0.74-2.33	0.359	Random	18.38,83.7 %,0.000
	**Stage I-II**	6	446	3.30	1.37-7.96	*0.008*	Random	45.94,89.1 %,0.000
	**Stage I-III/IV**	4	881	1.51	0.92-2.47	0.102	Random	7.75,761.3 %,0.051
	**Non-ADC**	2	184	1.88	0.88-4.01	0.105	Fixed	-
**DFS**	**All study**	8	1326	2.21	1.43-3.43	*0.000*	Random	28.35,75.3 %,0.000
	**Asian**	5	591	2.78	1.78-4.34	*0.000*	Random	4.67,14.4 %,0.323
	**Non-Asian**	3	735	1.83	1.09-3.06	*0.022*	Random	48.95,77.7 %,0.01
	**Stage I**	3	293	4.31	2.37-7.84	*0.000*	Fixed	0.79,0.0 %,0.672
	**Stage I-II**	2	265	1.51	1.02-2.23	*0.038*	Fixed	0.48,0.0 %,0.486
	**Stage I-III/IV**	3	783	2.02	0.97-4.20	0.06	Random	11.69, 82.9 %, 0.0.06

*Abbreviation:* ADC adenocarcinoma, CI confidence interval, DFS disease-free survival, Fixed, Fixed, Inverse Variance model, H Heterogeneity, HR hazard ratio, I^2^ I-squared, OS overall survival, Random, Random, I-V heterogeneity model

Next, subgroups including TNM stage (eight studies for stage I, eight for stages I–II, seven for stages I–III, and one for stages III–IV) and type of NSCLC (10 studies for ADC and two for non–ADC) were analyzed. The analyses indicated that high Ki–67 expression was associated with a shorter OS in stage I, stages I–II, and stages I–III patients (HR 1.85, 95 % CI 1.27–2.69, *P* = 0.001; HR 1.72, 95 % CI 1.20–2.46, *P* = 0.003; and HR 1.60, 95 % CI 1.21–2.12, *P* = 0.001, respectively) with heterogeneity (I^2^ = 78.7 %, *P* < 0.001; I^2^ = 76.1 %, *P* < 0.001; and I^2^ = 36.5 %, *P* = 0.001, respectively), but no association with shorter OS was observed in patients in stages III–IV (HR 1.31, 95 % CI 0.68–2.53, *P* = 0.42).

Another subgroup analysis (ADC vs. non–ADC) demonstrated that the ADC group showed a significant association between high Ki–67 expression and shorter OS (HR 2.21, 95 % CI 1.38–3.50, *P* < 0.001). However, the association was not significant in the non-ADC group (HR 1.88, 95 % CI 0.88–4.01, *P* = 0.105). Additionally, only Asian patients (vs. non-Asian patients) and the early-stage group (stages I–II vs. advanced stage) in the ADC group demonstrated significant associations between high Ki–67 expression and shorter OS. The combined HRs were 3.01, 95 % CI 1.96–4.02, *P* < 0.001 and 3.30, 95 % CI 1.37–7.96, *P* = 0.008, respectively. Non-Asian ADC patients and ADC patients at advanced stages of the disease showed no significant association between high Ki–67 expression and OS (HR 1.88, 95 % CI 0.88–4.01, *P* = 0.359 and HR 1.51, 95 % CI 0.92–2.47, *P* = 0.102, respectively).

### Correlation of high Ki-67 expression with OS in NSCLC using different cut-off values

Subgroup analysis demonstrated that the risks between Ki–67 expression and OS were not significant using different Ki-67 cut–off values (10 %, 25 %, 50 %). The pooled HRs and 95 % CIs were as follows: 1.80 (95 % CI 1.20–2.70) vs. 1.53 (95 % CI 1.28–1.84) for a cut–off value of 10 %, 1.57 (95 % CI 1.27–1.95) vs. 1.60 (95 % CI 1.22–2.08) for a cut–off value of 25 %, and 1.56 (95 % CI 1.30–1.86) vs. 1.72 (95 % CI 1.27–2.33) for a cut–off value of 50 % with significant heterogeneities (Additional file 4: Table S2, Additional file 5: Figure S1, Additional file 6: Figure S2 and Additional file 7: Figure S3).

### Correlation between high Ki-67 expression and DFS in NSCLC

**Fig. 3:**
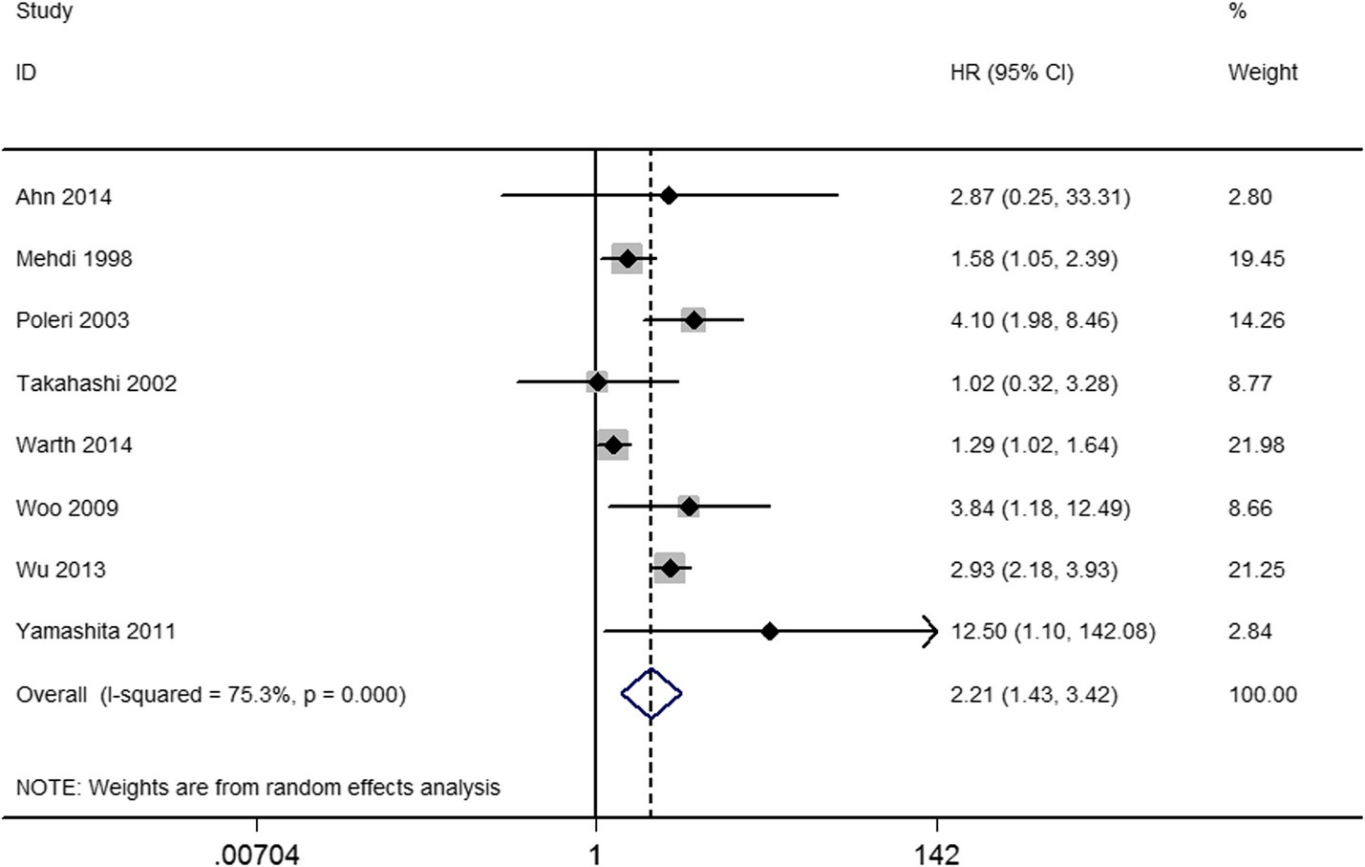
The hazard ratio (HR) of Ki-67 expression associated with DFS in all NSCLC patients. HR > 1 implied worse OS for the group with high Ki-67 expression

The pooled HR and 95 % CI for DFS provided in eight studies was 2.21, 95 % CI 1.43–3.43, *P* < 0.001, with heterogeneity (I^2^ = 75.3 %, *P* < 0.001; Fig. 3, Table 2). Subgroup analysis showed that the risk in Asian patients was higher than that in non-Asian patients, and the combined HRs and 95 % CIs were as follows: HR 2.78, 95 % CI 1.78–4.34, *P* < 0.001 and HR 1.83, 95 % CI 1.09–3.06, *P* = 0.022, respectively. Further subgroup analysis indicated that the very early stage (stage I) showed the highest risk, when compared with stages I–II or I–III, with the following combined HRs and 95 % CIs: HR 4.31, 95 % CI 2.37–7.84, *P* < 0.001; HR 1.51, 95 % CI 1.02–2.23, *P* = 0.038; and HR 2.02, 95 % CI 0.97–4.20, *P* < 0.06, respectively.

### Association between high Ki-67 expression and the clinicopathological characteristics of NSCLC

In this meta-analysis, clinicopathological features, such as age, gender, smoking habits, pathological type, lymph node status, and tumor differentiation grade, as impacted by increased Ki-67 expression were compared on the basis of the 32 studies. The results of the meta-analysis showed significant associations between high Ki-67 expression and being male, smoking habits, being a non-ADC patient, higher tumor stage (T_2-4_) and poorer differentiation grade (moderate or poor); the combined ORs and 95 % CIs were as follows: OR 1.89, 95 % CI 1.53–2.33, *P* < 0.001; OR 2.20, 95 % CI 1.72–2.82, *P* < 0.000; OR 1.88, 95 % CI 1.60–2.22, *P* < 0.001; OR 1.46, 95 % CI 1.13–1.88, *P* = 0.004; and OR 1.47, 95 % CI 1.15–1.88, *P* = 0.002, respectively. Moreover, significant associations between Ki–67 and gender (male), being a non-ADC patient, higher tumor stage, and poorer differentiation were seen only in Asian NSCLC patients. The combined ORs and 95 % CIs were as follows: OR 2.18, 95 % CI 1.67–2.81, *P* < 0.001; OR 2.22, 95 % CI 1.82–2.70; OR 1.47, 95 % CI 1.12–1.94, *P* = 0.006; and OR 1.50, 95 % CI 1.15–1.94, *P* = 0.002, respectively (Table 3).

**Table 3:** OR values for NSCLC subgroups according to clinical characteristics

Outcome of interest	Studies	Patients	OR	95%CI	P value	Model	H, I^2^, P value
**Age (>60 vs. <60)**	n = 6	1531	1.08	0.85-1.37	0.553	Fixed	5.37,6.9 %,0.37
**Gender (Male vs. Female)**	n = 11	2696	1.89	1.53-2.33	*0.000*	Fixed	10.52,5.0 %,0.40
Asian	n = 8	1933	2.18	1.67-2.81	*0.000*	Fixed	5.97,0.0 %,0.54
Non-Asian	n = 3	763	1.38	0.96-1.99	0.084	Fixed	0.97,0.0 %,0.61
**Smoke habits** **(Smoke vs. Non-smoke)**	n = 7	1785	2.20	1.72-2.82	*0.000*	Fixed	4.77,0.0 %,0.57
**Non-ADC vs. ADC**	n = 15	3185	1.88	1.60-2.22	*0.000*	Fixed	17.13,18.3 %,0.25
Asian	n = 10	2345	2.22	1.82-2.70	*0.000*	Fixed	3.81,0.0 %,0.92
Non-Asian	n = 5	840	1.31	0.98-1.75	0.073	Fixed	4.74,15.6 %,0.32
**T**_**2-4**_ **vs T**_**1**_	n = 9	2156	1.46	1.13-1.88	*0.004*	Fixed	3.13,0.0 %,0.93
Asian	n = 7	1938	1.47	1.12-1.94	*0.006*	Fixed	2.86,0.0 %,0.83
Non-Asian	n = 2	218	1.37	0.71-2.65	0.349	Fixed	0.23,0.0 %,0.63
**N**_**1-3**_ **vs N**_**0**_	n = 11	2443	1.01	0.83-1.22	0.927	Fixed	10.73,6.8 %,0.38
**Differentiation (well vs. moderate/poor)**	n = 9	2029	1.47	1.15-1.88	*0.002*	Fixed	6.04,0.0 %,0.64
Asian	n = 7	1837	1.50	1.15-1.94	*0.002*	Fixed	5.14,0.0 %,0.53
Non-Asian	n = 2	192	1.28	0.60-2.74	0.517	Fixed	0.81,0.0 %,0.37

*Abbreviation:* ADC adenocarcinoma, CI confidence interval, Fixed, Fixed, Inverse Variance model, H Heterogeneity, I^2^ I-squared, OR,odds Ratio

There was no significant association between Ki–67 expression and age (>60 vs. < 60) or lymph node status (N_1–3_ vs. N_0_); the combined ORs and 95 % CIs were OR 1.08, 95 % CI 0.85–1.37, *P* = 0.553 and OR 1.01, 95 % CI 0.83–1.22, *P* = 0.927, respectively (Table 3).

### Sensitivity analysis

Sensitivity analysis showed that the pooled HRs of OS and DFS were similar to those calculated after one study was removed and the rest were reanalyzed (Additional file 8: Figure S4 and Additional file 9: Figure S5). Moreover, the HR remained unchanged (HR 1.86, 95 % CI 1.44–2.28, *P* < 0.001 and HR 2.74, 95 % CI 1.25–4.22, *P* < 0.001, respectively) after the ‘trim and fill’ method was used (Additional file 10: Figure S6 and Additional file 11: Figure S7). Additionally, we report the combined HR and 95 % CI results of the fixed effects model: pooled HR 1.86, 95 % CI 1.44–2.28, *P* < 0.001 for OS and pooled HR 1.52, 95 % CI 1.08–1.96, *P* < 0.001 for DFS. These values were consistent with the random-effects model. Both analyses support the reliability of our results.

### Publication bias

**Fig. 4:**
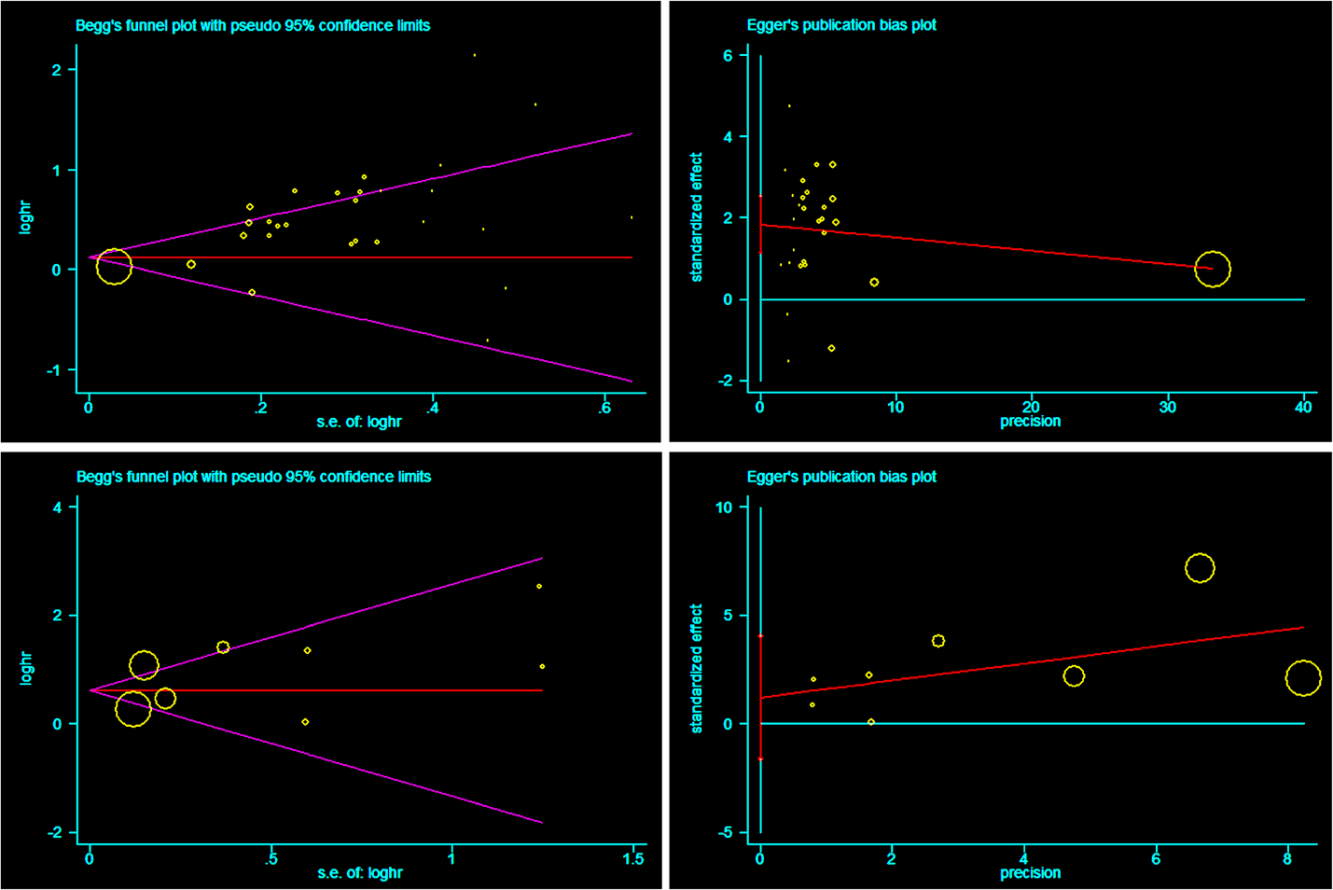
Funnel Plots of Begg’s and Egger’s were used to detect publication bias on OS and DFS. Begg’s funnel plots showed seemingly publication bias on OS (A) while Egger’s funnel plots showed no publication bias on OS in all NSCLC. It showed no publication bias on DFS in Begg’s test (C) and Egger’s test (D)

Begg’s test indicated no publication bias among the studies included in the current meta-analysis regarding the HRs of OS and DFS, with *P* values of 0.395 and 0.902, respectively. Egger’s test indicated no publication bias for DFS (*P* = 0.34), but it showed seemingly significant publication bias for OS after assessing the funnel plot (*P* < 0.001; Fig. 4).

## Discussion

Ki-67 is a nuclear non-histone protein first identified 30 years ago [2]. Because it is expressed during all phases of the cell cycle except the resting stage (G0), it has been used as a marker to evaluate proliferation in NSCLC [5, 9, 33, 44], as well as in other tumors, such as lymphoma [13], esophageal cancer [49], breast cancer [10], and prostate cancer [50]. Nonetheless, studies examining the relationship between Ki-67 expression and NSCLC prognosis have been inconsistent [33, 35, 42, 45].

Meta-analytic techniques using non-randomized controlled trials (NRCTs) may be useful in certain clinical settings where the number or the sample size of the RCTs is insufficient [48]. The results of the current meta-analysis revealed that high Ki-67 expression in patients with NSCLC *was* associated with a poorer prognosis for OS (HR 1.59, 95 % CI 1.35–1.88, *P* < 0.001), consistent with a previous meta-analysis, published in 2004 [2], but in this case with nearly three–fold as many patients and double the number of studies. In addition, it was first reported that high Ki-67 expression in NSCLC patients was associated with a poor survival outcome for DFS (combined HR 2.21, 95 % CI 1.43–3.43, *P* < 0.001). Sensitivity analysis suggested that the association between high Ki-67 expression and NSCLC prognosis was stable and unchanged after removing any one study. Also, the results of the current meta-analysis show that high Ki-67 expression was more common in males (OR = 1.89, *P* < 0.001), smokers (OR = 2.20, *P* < 0.001), those in later tumor stages (OR = 1.46, *P* = 0.004), or those with poorer differentiation (OR = 1.47, *P* = 0.002), which has been linked to more aggressive tumors. Overall, the results of the current meta-analysis suggest that increased Ki-67 expression exerts a significantly adverse effect on the prognosis of NSCLC patients. To our knowledge, this study is the most comprehensive and detailed meta-analysis to evaluate the association between Ki-67 expression and survival in NSCLC patients.

NSCLC is a malignancy displaying substantial heterogeneity, and the clinical and biological characteristics of the different subtypes of NSCLC vary substantially [51]. In this meta-analysis, high Ki-67 expression was a valuable indicator both of OS and DFS for ADC; this is consistent with the latest large-scale study conducted by Warth and colleagues, which included 1482 patients [47]. Furthermore, higher Ki-67 expression was a more valuable indicator for early (stages I–II) NSCLC and early (stages I–II) ADC. However, it showed no association between survival and being a non-ADC patient, with a HR of 1.88 and a 95 % CI of 0.88–4.01 for OS. Due to the strict inclusion criteria, only two studies in the current meta-analysis were included, and several studies without enough survival data were excluded. However, several types of non-ADC including squamous cell carcinoma (SQCC), large cell lung carcinoma (LCC), and bronchial gland carcinoma (BGC) may make it difficult to obtain reliable results. The association between high Ki-67 expression and survival outcome in non-ADC patients still requires further investigation.

It was reported that Asian ethnicity is a favorable prognostic factor for OS in NSCLC and is independent of smoking status [52, 53]. However, no data regarding the impact of Ki-67 and race/ethnicity on the outcome of NSCLC patients are available. Subgroup analysis in this study showed that higher Ki-67 expression indicated a poorer outcome in Asian NSCLC patients compared with non-Asian patients (HR 1.97, 95 % CI 1.43–2.71 vs. HR 1.37, 95 % CI 1.15–1.64 for OS and HR 2.78, 95 % CI 1.78–4.34 vs. HR 1.83, 95 % CI 1.09–3.06 for DFS). To date, there has been no consensus regarding the significance of Ki-67 in NSCLC in Asian versus non-Asian NSCLC patients. In the current study, a strong relationship was established between poor prognostic indicators and Ki-67 expression only in Asian patients. In addition, high Ki-67 expression was associated with larger tumor size and differentiation, which is in line with previous studies [2]. Furthermore, we found higher Ki-67 expression levels in Asian patients compared with non-Asian patients (31.39 % vs. 26.77 %, Additional file 12: Table S3), whereas no positive patients/total patients ratio differences were demonstrated (44.91 % vs. 47.18, *P* = 711). Therefore, the alteration of Ki-67 expression may contribute to the differences in the tumor biology observed between Asian and non-Asian patients with NSCLC. Although, future validation and investigations are needed, these data may provide new insights into biological aggressiveness of NSCLC in Asian versus in non-Asian patients.

Heterogeneity was significant in this meta-analysis, and it could not be ruled out by using a random-effects model or multiple subgroup analyses. For reasons of homogeneity, we analyzed only the studies dealing with NSCLC histology and restricted the analysis to the histological subtypes or tumor stages for which we had sufficient numbers of studies. Furthermore, the technique(s) used to identify the expression of Ki-67 can be a potential source of bias. The use of different antibodies (anti-Ki67 mAb or anti-MIB-1 mAb) and a protocol to count the number of cells stained by these antibodies without a received standard antibody concentration may yield variation among the studies. Moreover, the cut-off value used to define a tumor with ‘positive’ Ki-67 staining is often arbitrary and varies according to the investigator, from a low percentage to more than 50 %. Martin *et al.* [2] introduced two cut-off levels for defining Ki-67 expression in tumors, one to exclude patients with slowly proliferating tumors due to chemotherapeutic protocols (10 %) and one to identify patients sensitive to chemotherapy protocols (25 %). In addition, Warth *et al.* introduced 50 % as the cut-off value for defining Ki-67 expression in SQCC [47]. In this study, the adverse effect of high Ki-67 expression on OS showed similar results using these three recommended cut-off values (Additional file 4: Table S2, Additional file 5: Figure S1, Additional file 6: Figure S2 and Additional file 7: Figure S3). Nonetheless, a consensus for the optimal cut-off value for Ki-67 needs to be reached and validated in NSCLC patients in future studies.

It is important to note that the current study encountered difficulties, similar as most meta-analysis. First, it was based on summary data rather than data from individual patients. Therefore, multivariate analyses for confounding factors such as histological subtypes, gender and smoking status were not performed. A meta-regression model that adjusted for those factors that were found to be correlated with high K-67 levels was also not performed, too. Second, our search was limited to published studies and excluded unpublished trials or results in abstract form, which may have led to publication bias. Third, the unequal number of studies from Asian countries, with data derived mainly from Japan, China, and Korea may have also been a source of bias. Hence, our analysis may reflect outcomes from East Asia rather than from Asia in general.

There are several advantages to this study. First, large numbers (32 studies and 5600 patients) were analyzed, whereas only 17 studies and 1863 NSCLC patients were considered in the meta-analysis published in 2004. Second, important clinical parameters including age, gender, tumor stage, histology, and race/ethnicity were included in the analysis. The association between high Ki-67 expression and DFS was also investigated. However, as several limitations still exist, the results need to be interpreted cautiously. First, the number of included studies and included NSCLC patients were relatively small. Moreover, heterogeneity was inevitable among the groups due to the impossibility of matching patient characteristics across all studies. This may have weakened the results to some extent. Second, publication bias was unavoidable for clinical evidence, because the relevant data were extracted from non-randomized controlled trials. Third, reports in languages other than English were excluded; therefore, potential language bias may have been present. Lastly, some data for OS were extracted from survival curves or other available data rather than provided directly. Although the method used for extrapolation of HRs and 95 % CIs is widely accepted and we did not identify any major differences in current study (Additional file 13: Figure S8), we could not completely eliminate inaccuracy in the extracted survival rates.

## Conclusions

Despite these limitations, our systematic review of the literature showed that high expression of Ki-67 in NSCLC patients, particularly during the early stages (stages I–II), in Asians, and in ADC patients is a poor prognostic indicator for survival outcome. Further adequately designed prospective studies with standardization of the immunohistochemistry technique, especially standardization of the cut-off threshold value, need to be conducted to confirm these results.

## Additional files

Additional file 1:
**PRISMA 2009 checklist in current meta-analysis.**

Additional file 2: **Based on the editor's work.** PRISMA 2009 Flow Diagram in current meta-analysis.
Additional file 3: Table S1. Definitions of 18 items of study reporting quality.
Additional file 4: Table S2. HR values of OS of NSCLC subgroups depended on cutoff value.
Additional file 5: Figure S1. The hazard ratio (HR) of Ki-67 expression associated with overall survival (OS) in all NSCLC patients subgroup (cutoff value >10 % and cutoff value <10 %).
Additional file 6: Figure S2. The hazard ratio (HR) of Ki-67 expression associated with overall survival (OS) in all NSCLC patients subgroup (cutoff value >25 % and cutoff value <25 %).
Additional file 7: Figure S3. The hazard ratio (HR) of Ki-67 expression associated with overall survival (OS) in all NSCLC patients subgroup (cutoff value >50 % and cutoff value <50 %).
Additional file 8: Figure S4. Sensitivity analysis of all the studies assessing OS.
Additional file 9: Figure S5. Sensitivity analysis of all the studies assessing DFS.
Additional file 10: Figure S6. Trim and fill analysis of all the studies assessing OS.
Additional file 11: Figure S7. Trim and fill analysis of all the studies assessing DFS.
Additional file 12: Table S3. Comparisons of Ki-67 expression in NSCLC between Asian and Non-Asian Studies.
Additional file 13: Figure S8. The hazard ratio (HR) of Ki-67 expression associated with OS in all NSCLC patients subgroup (NA: HR and 95%CI extracted from articles; R: HR and 95%CI provided directly).

## Abbreviations

LC: Lung cancer
NSCLC: Non-small cell lung cancer
ADC: Adenocarcinoma
BGC: Bronchial gland carcinoma
PRISMA: Preferred Reporting Items for Systematic Reviews and Meta-Analyses
IHC: Immunohistochemistry
OS: Overall survival
DFS: Disease-free survival
HR: Hazard ratio
CI: Confidence interval
OR: Odds ratio
