# Supplementary material for: Ki-67 as a prognostic marker in early-stage non-small cell lung cancer in Asian patients: a meta-analysis of published studies involving 32 studies
Source: BMC Cancer. 2015 Jul 15;15:520. doi: 10.1186/s12885-015-1524-2 (PMC4502553; doi:10.1186/s12885-015-1524-2)
Supplement: Additional file 2: — Based on the editor's work. PRISMA 2009 Flow Diagram in current meta-analysis. [file 12885_2015_1524_MOESM2_ESM.doc]

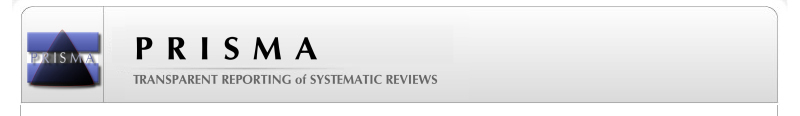
**PRISMA 2009 Flow Diagram**

**Screening**

**Included**

**Eligibility**

**Identification**

Records identified through database searching
(n = 2046 )

Additional records identified through other sources
(n = 0 )

Records after duplicates removed
(n =200 )

Records screened
(n = 1846 )

Records excluded
(n =1809 )

Full-text articles assessed for eligibility
(n =37 )

Full-text articles excluded, with reasons
(n = 7 )

Studies included in qualitative synthesis
(n = 30 )

Studies included in quantitative synthesis (meta-analysis)
(n = 32 )
