# Supplementary material for: Ki-67 as a prognostic marker in early-stage non-small cell lung cancer in Asian patients: a meta-analysis of published studies involving 32 studies
Source: BMC Cancer. 2015 Jul 15;15:520. doi: 10.1186/s12885-015-1524-2 (PMC4502553; doi:10.1186/s12885-015-1524-2)
Supplement: Additional file 3: Table S1. — Definitions of 18 items of study reporting quality. [file 12885_2015_1524_MOESM3_ESM.doc]

Table 1. Definitions of 18 items of study reporting quality

| **Study design**  1. Objectives or pre-specified hypothesis: state the study objectives, pre-specified hypothesis or study protocol  2. Sample size: state a statistical sample size or power calculation  3. Follow-up description: state the follow-up period or the median follow-up time  4. Population source: state health care setting from which patients were recruited  5. Population selection criteria: state inclusion or exclusion  6. Population characteristics: state the population characteristics (e.g., age, gender, and disease stage)  7. Number of patients included in each stage of the analysis and reason for dropout: description of number of patients at different stage, including the number of patients who participate in the study, who met the inclusion criteria, and who followed up and reason for dropout |
| --- |
| **Assay method**  1. Sample handling: state the method of storage  2. Assay method: state the type of assay method used to measure Ki-67  3. Manufacturer: state the name of company which makes the assay for Ki-67  4. Cutoff point determination: state methods used for cutoff point determination |
| **Confounders**  1. Conventional risk factors: state the conventional risk factors (e.g., age, gender, depth of tumor, lymph node metastasis)  2. Other biomarkers (e.g., p53, RB or microvessel density, etc. ): state other biologic marker relating with the disease |
| **Outcome**  1. Clinical endpoint: define the clinical endpoint  2. Validation: state the outcome events checked by independent source (e.g., medical records, outpatient visits, by letter, and by telephone) |
| **Analysis**  1. Univariate estimate: report the effect of Ki-67 on outcome  2. Multivariate estimate: adjusted for risk factors or other biomarkers (list above)  3. Missing value: state the number of patients with missing value for Ki-67 or confounders and how to deal with it |

Noted: A study can be award a maximum of 2 points (possible values 2, 1, and 0: 2 represented the complete description, 1 represented partly matched descripted, 0 represented no matched description) and the maximum score was 36.
