# Supplementary material for: Ki-67 as a prognostic marker in early-stage non-small cell lung cancer in Asian patients: a meta-analysis of published studies involving 32 studies
Source: BMC Cancer. 2015 Jul 15;15:520. doi: 10.1186/s12885-015-1524-2 (PMC4502553; doi:10.1186/s12885-015-1524-2)
Supplement: Additional file 4: Table S2. — HR values of OS of NSCLC subgroups depended on cutoff value. [file 12885_2015_1524_MOESM4_ESM.doc]

**Supplement Table 2.** HR values of OS of NSCLC subgroups depended on cutoff value

| **Cutoff Value (%)** | **Studies** | **HR** | **95%CI** | **P value** | **Model** | **H, I^2^, P value** |
| --- | --- | --- | --- | --- | --- | --- |
| **<10** | n=7 | 1.80 | 1.20-2.70 | 0.005 | Random | 10.58,52.7%,0.060 |
| **≥10** | n=21 | 1.53 | 1.28-1.84 | 0.000 | Random | 10.52,76.3%，0.000 |
| **<25** | n=19 | 1.57 | 1.27-1.95 | 0.000 | Random | 83.08,78.3%,0.000 |
| **≥25** | n=8 | 1.60 | 1.22-2.08 | 0.001 | Random | 14.09,50.3%,0.05 |
| **<50** | n=24 | 1.56 | 1.30-1.86 | 0.000 | Random | 96.15,76.1%,0.000 |
| **≥50** | n=3 | 1.72 | 1.27-2.33 | 0.000 | Fixed | 0.77,0.0%,0.682 |

**Abbreviation:** Fixed, Fixed, Inverse Variance model; H, Heterogeneity; HR, hazard ratio; I^2^, I-squared; Random,

Random, I-V heterogeneity model.
