# Supplementary figures and images for: Ki-67 as a prognostic marker in early-stage non-small cell lung cancer in Asian patients: a meta-analysis of published studies involving 32 studies
Source: BMC Cancer. 2015 Jul 15;15:520. doi: 10.1186/s12885-015-1524-2 (PMC4502553; doi:10.1186/s12885-015-1524-2)

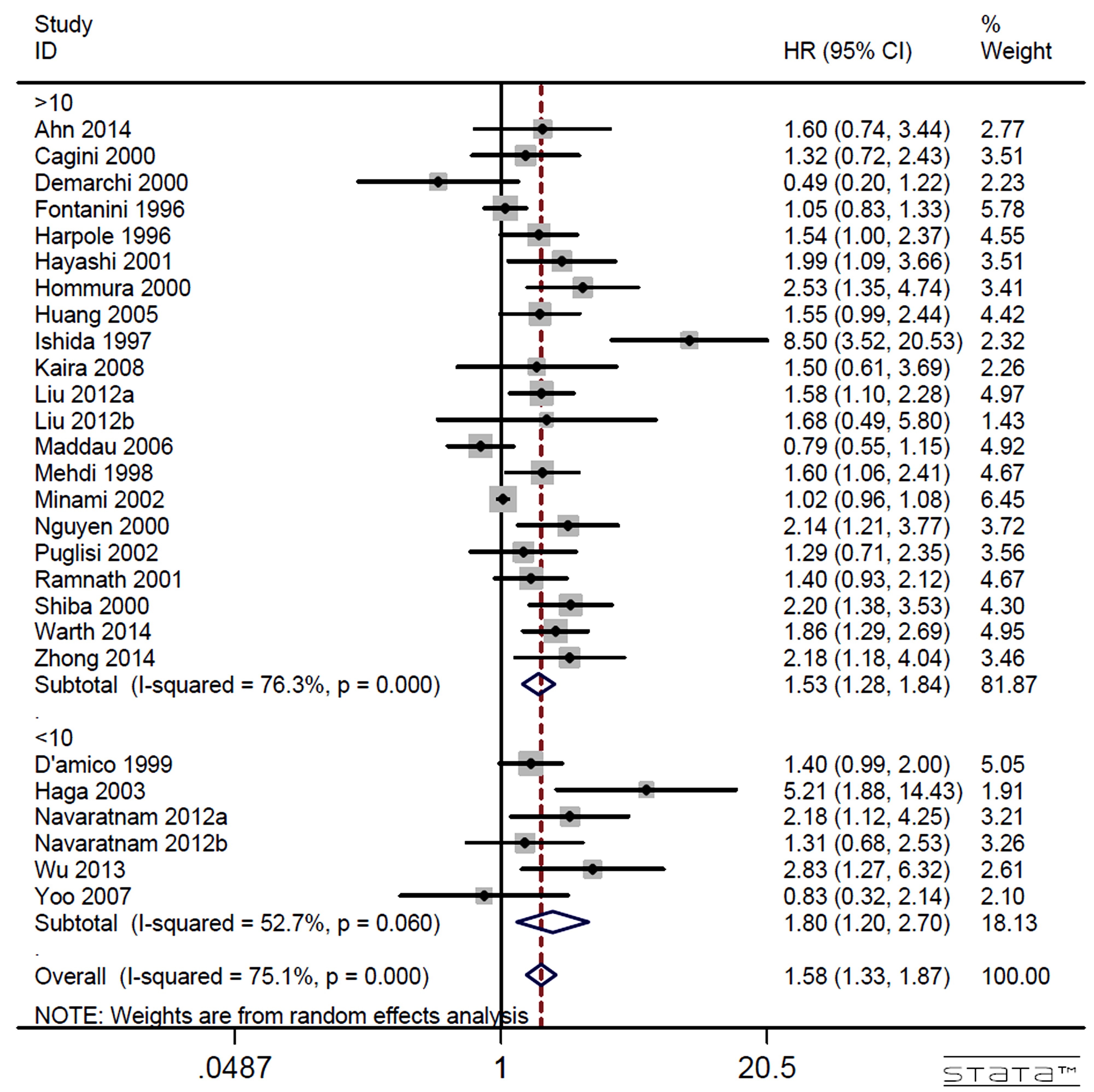

Supplement: Additional file 5: Figure S1. — The hazard ratio (HR) of Ki-67 expression associated with overall survival (OS) in all NSCLC patients subgroup (cutoff value >10 % and cutoff value <10 %). [file 12885_2015_1524_MOESM5_ESM.tiff]

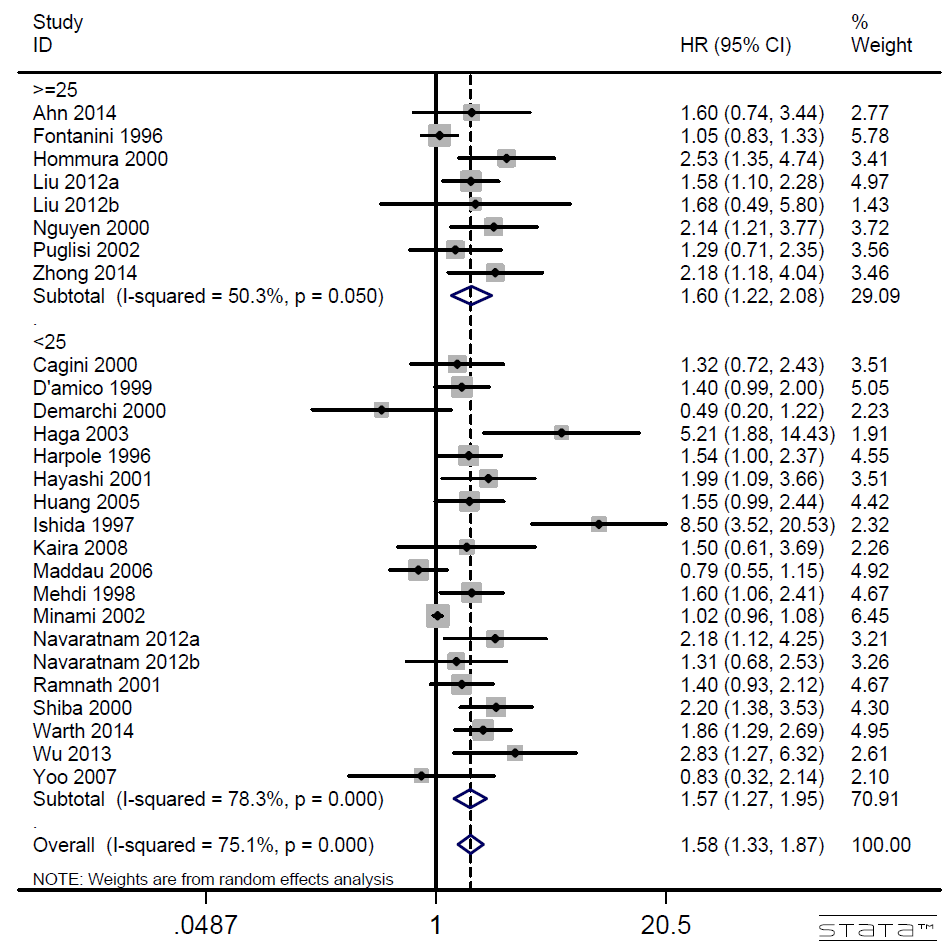

Supplement: Additional file 6: Figure S2. — The hazard ratio (HR) of Ki-67 expression associated with overall survival (OS) in all NSCLC patients subgroup (cutoff value >25 % and cutoff value <25 %). [file 12885_2015_1524_MOESM6_ESM.tiff]

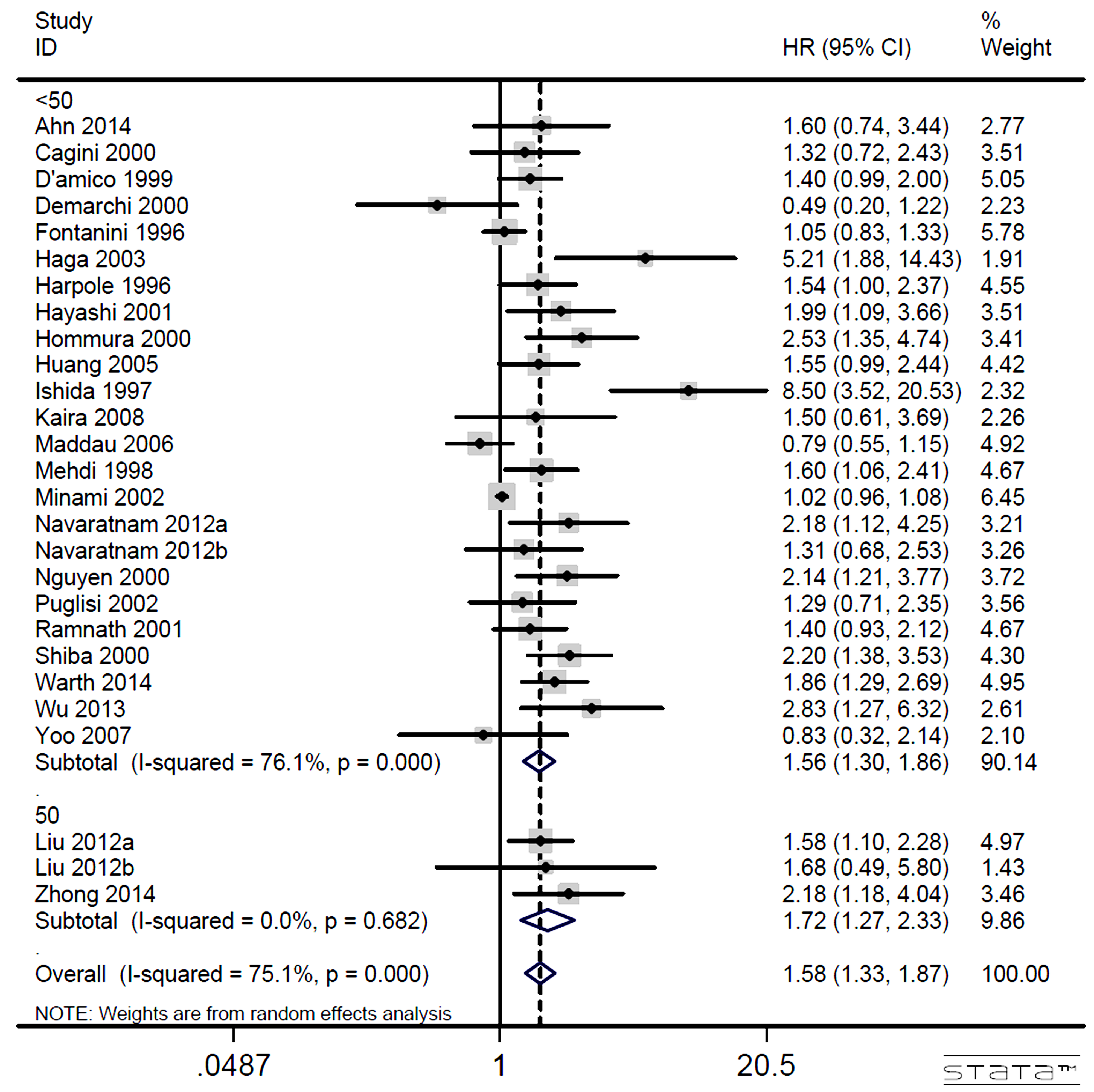

Supplement: Additional file 7: Figure S3. — The hazard ratio (HR) of Ki-67 expression associated with overall survival (OS) in all NSCLC patients subgroup (cutoff value >50 % and cutoff value <50 %). [file 12885_2015_1524_MOESM7_ESM.tiff]

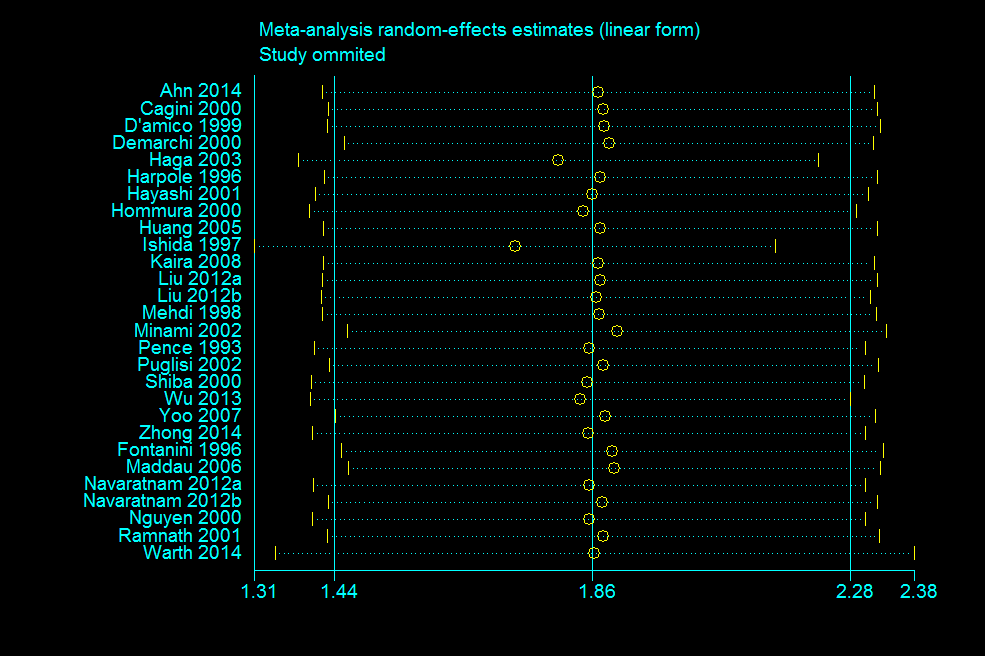

Supplement: Additional file 8: Figure S4. — Sensitivity analysis of all the studies assessing OS. [file 12885_2015_1524_MOESM8_ESM.tiff]

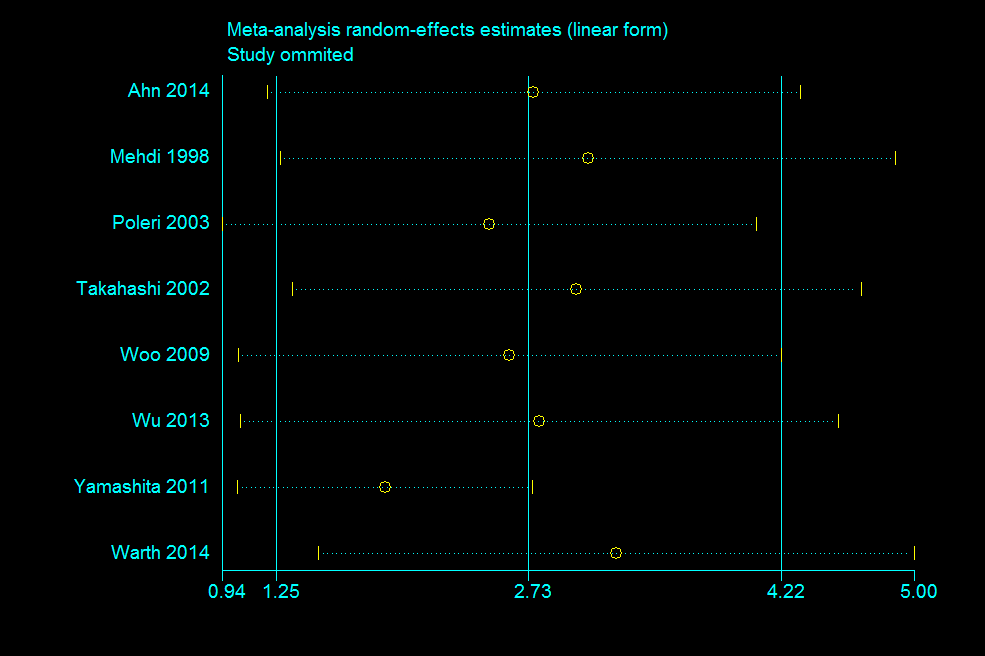

Supplement: Additional file 9: Figure S5. — Sensitivity analysis of all the studies assessing DFS. [file 12885_2015_1524_MOESM9_ESM.tiff]

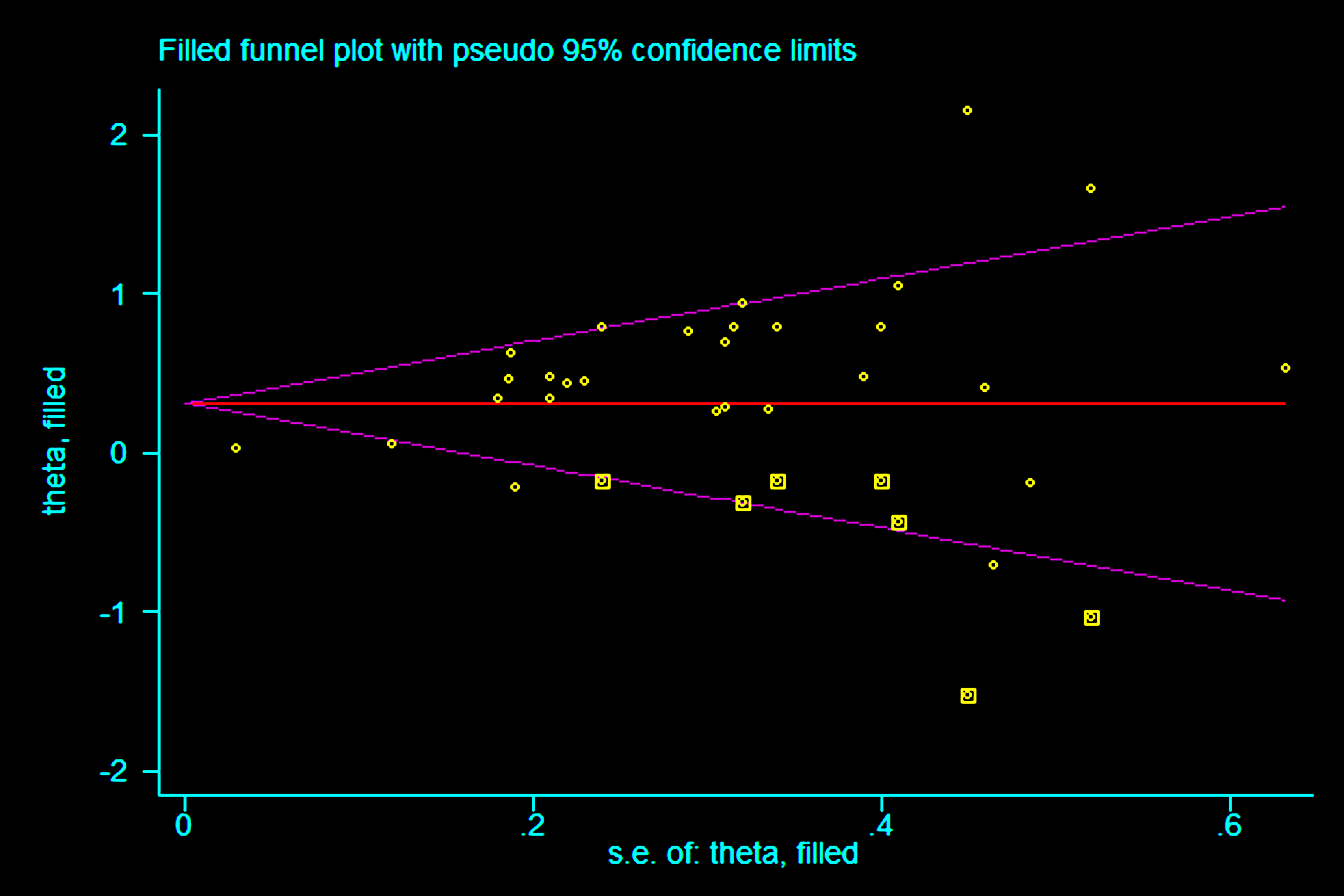

Supplement: Additional file 10: Figure S6. — Trim and fill analysis of all the studies assessing OS. [file 12885_2015_1524_MOESM10_ESM.tiff]

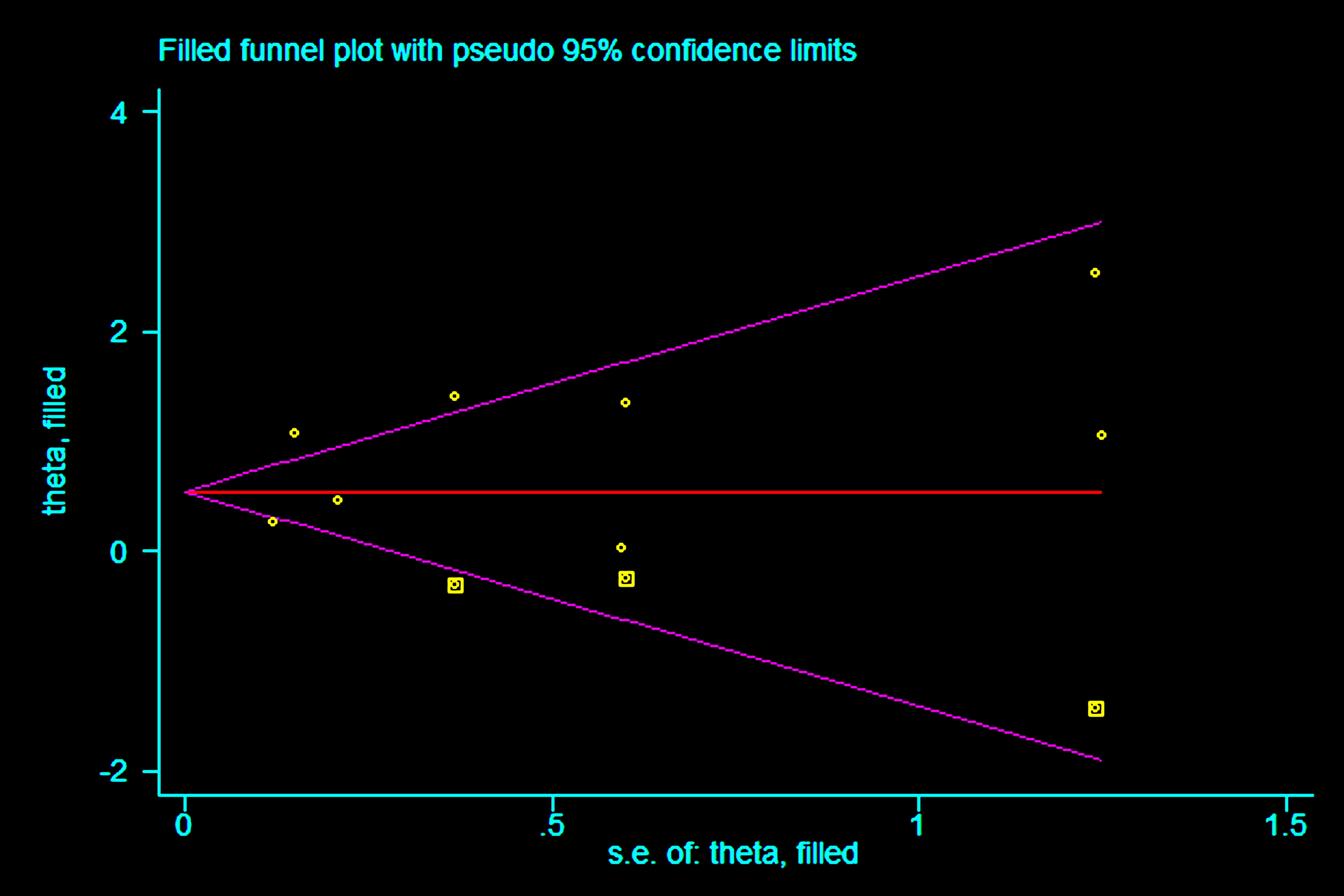

Supplement: Additional file 11: Figure S7. — Trim and fill analysis of all the studies assessing DFS. [file 12885_2015_1524_MOESM11_ESM.tiff]

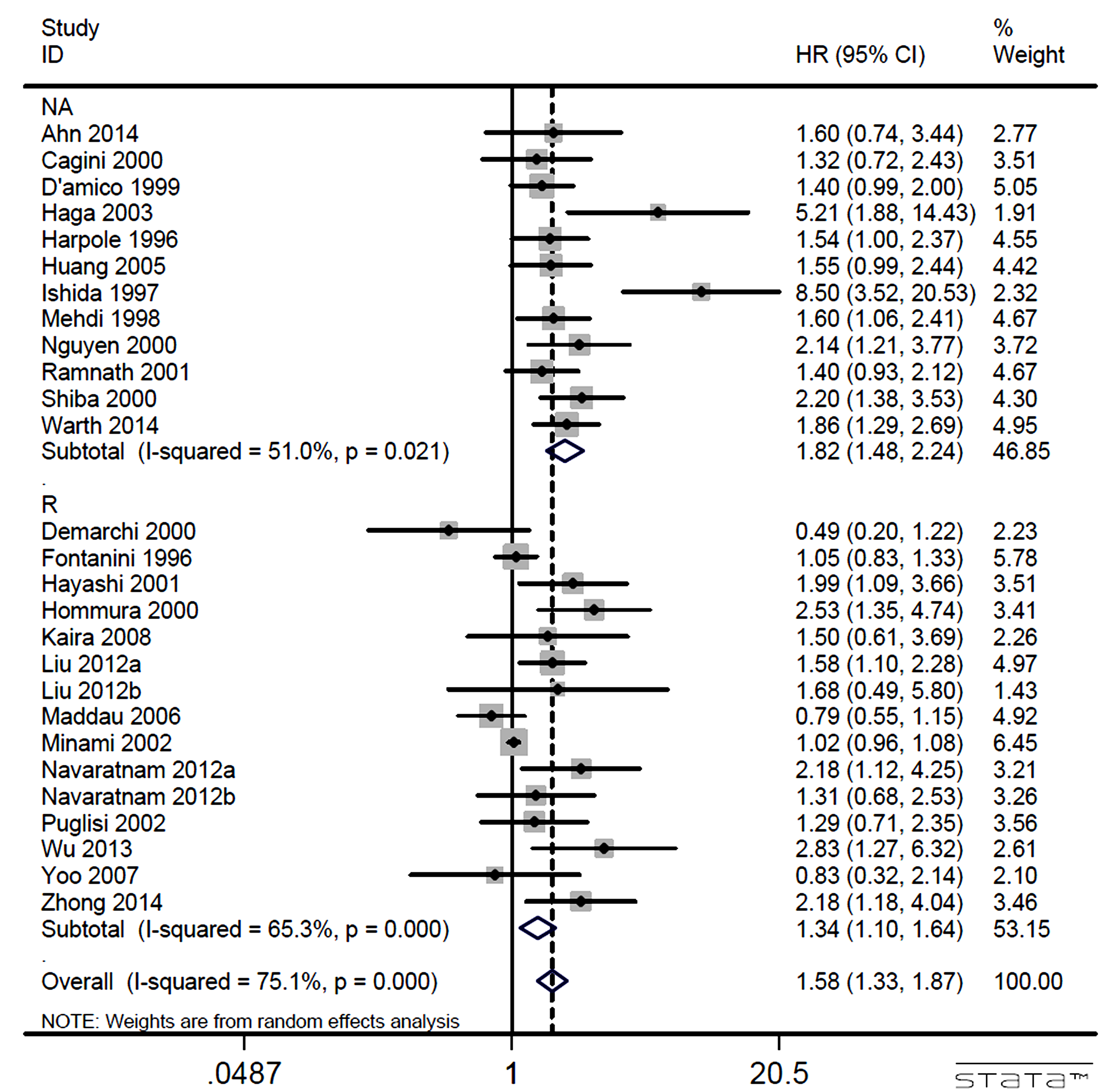

Supplement: Additional file 13: Figure S8. — The hazard ratio (HR) of Ki-67 expression associated with OS in all NSCLC patients subgroup (NA: HR and 95%CI extracted from articles; R: HR and 95%CI provided directly). [file 12885_2015_1524_MOESM13_ESM.tiff]
