# Supplementary material for: Ki-67 as a prognostic marker in early-stage non-small cell lung cancer in Asian patients: a meta-analysis of published studies involving 32 studies
Source: BMC Cancer. 2015 Jul 15;15:520. doi: 10.1186/s12885-015-1524-2 (PMC4502553; doi:10.1186/s12885-015-1524-2)
Supplement: Additional file 12: Table S3. — Comparisons of Ki-67 expression in NSCLC between Asian and Non-Asian Studies. [file 12885_2015_1524_MOESM12_ESM.doc]

**Supplement Table 3.** Comparisons of Ki-67 expression in NSCLC between Asian and Non-Asian Studies

|  | **Asian studies** | **Non-Asian studies** | ***P* Value** |
| --- | --- | --- | --- |
| **% Ki-67 positive**  **(Positive cases/Total cases)** | 44.91±18.57, n=14 | 47.18±12.95, n=14 | 0.711 |
| **Combined Ki-67 value** | 31.39±23.87,n=5 | 26.77±22.72, n=5 | - |

All values are expressed as mean ± SD. n, studies. Independent-samples *t* test was used, and Significant differences were accepted at *p* < 0.05.
